# Proton-Binding Sites of Acid-Sensing Ion Channel 1

**DOI:** 10.1371/journal.pone.0016920

**Published:** 2011-02-14

**Authors:** Hiroshi Ishikita

**Affiliations:** 1 Career-Path Promotion Unit for Young Life Scientists, Graduate School of Medicine, Kyoto University, Kyoto, Japan; 2 Precursory Research for Embryonic Science and Technology, Japan Science and Technology Agency, Saitama, Japan; University of Pennsylvania, United States of America

## Abstract

Acid-sensing ion channels (ASICs) are proton-gated cation channels that exist throughout the mammalian central and peripheral nervous systems. ASIC1 is the most abundant of all the ASICs and is likely to modulate synaptic transmission. Identifying the proton-binding sites of ASCI1 is required to elucidate its pH-sensing mechanism. By using the crystal structure of ASIC1, the protonation states of each titratable site of ASIC1 were calculated by solving the Poisson-Boltzmann equation under conditions wherein the protonation states of all these sites are simultaneously in equilibrium. Four acidic-acidic residue pairs—Asp238-Asp350, Glu220-Asp408, Glu239-Asp346, and Glu80-Glu417—were found to be highly protonated. In particular, the Glu80-Glu417 pair in the inner pore was completely protonated and possessed 2 H^+^, implying its possible importance as a proton-binding site. The p*K*_a_ of Glu239, which forms a pair with a possible pH-sensing site Asp346, differs among each homo-trimer subunit due to the different H-bond pattern of Thr237 in the different protein conformations of the subunits. His74 possessed a p*K*_a_ of ≈6–7. Conservation of His74 in the proton-sensitive ASIC3 that lacks a residue corresponding to Asp346 may suggest its possible pH-sensing role in proton-sensitive ASICs.

## Introduction

Acid-sensing ion channels (ASICs) are proton-gated cation channels that exist throughout the mammalian central and peripheral nervous systems. ASIC1a, ASIC1b, ASIC2a, ASIC2b, ASIC3, and ASIC4 have been already identified [1]. With the exception of acid-insensitive ASIC2b and ASIC4 when expressed as a homo-trimer [2], most ASICs are activated by a decrease in the extracellular pH, i.e. increase of proton concentration. Activation of these channels by protons plays an important role in physiological and pathological processes such as nociception, mechanosensation, synaptic plasticity, and acidosis-mediated neuronal injury [3]. Thus, ASICs are important pharmacological targets in neurological diseases.

**Figure 1:**
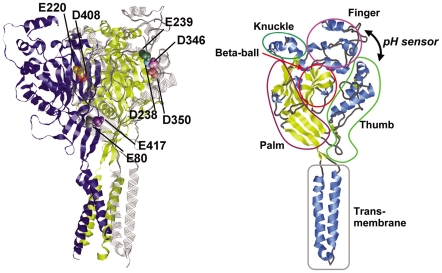
Overview of ASIC1 [5]. Acidic-acidic residue pairs (only in subunit A) are depicted as spheres. Subunit A is depicted as gray strands, and subunits B and C are depicted as blue and yellow ribbons, respectively.

In the central nervous system, ASIC1 is the most abundant of all the ASICs and is likely to modulate synaptic transmission, memory, and fear conditioning [4]. A recently identified crystal structure of ASIC1 from chicken at 1.9 Å resolution [5] reveals the geometry of a transmembrane domain, which comprises 2 transmembrane helices, and another 5 domains, namely, finger, thumb, knuckle, palm, and *β*-ball (Figure 1). The finger and thumb domains, located considerably away from the transmembrane domain, are of particular interest because their domain interface was proposed to be a possible pH-sensing site of ASIC1, based on the observations of the crystal structure and mutational studies by Jasti et al. [5] (Figure 1). The following pH-sensing mechanism wherein Asp346 plays a key role has been proposed: protonation/deprotonation of Asp346 of the thumb domain could alter the interaction with its pair partner Glu239 on the finger domain, resulting in the translocation of the thumb domain. Since the thumb domain is linked with the transmembrane domain, movement of the thumb domain causes reorientation of the transmembrane domain and modifies channel gating (see supplementary information: http://www.nature.com/nature/journal/v449/n7160/extref/nature06163-s2.htm in Ref. [5]). On the other hand, other residues have also been proposed to play an important role in pH sensitivity, mainly by mutational studies. In ASIC2a, mutation of a residue that corresponds to His74 of ASIC1 resulted in pH-sensing deficiency [6], [7]. The residue pair Asp79-Glu80 is conserved in all pH-sensitive ASICs. Mutations of the corresponding residue pair in ASIC3 enhance the rate of channel inactivation [8] and those in ASIC2a render the channels proton insensitive [7]. To identify the p*K*_a_ values or the protonation states of these residues is, therefore, a starting point in an effort to understand the pH-sensing mechanism of proton-sensitive ASICs.

In the present study, by using the ASIC1 crystal structure [5] and solving the linear Poisson-Boltzmann (LPB) equation, the protonation probabilities of all the titratable sites identified in the crystal structure (51 Arg, 75 Asp, 101 Glu, 73 Lys, 72 Tyr, 15 His, and 3 pairs of N/C-terminal residues) were calculated. In this system, the protonation states of each titratable site are affected by the protonation states of all the titratable sites, accomplishing a completely equilibrated system in terms of the protein protonation pattern. The protonation states of the residues and their p*K*_a_ values were obtained and compared among the homo-trimer subunits A, B, and C of ASIC1. In the present study, the computational conditions and procedures employed in previous studies on other ion [9], [10] and proton [11], [12] channels were consistently used.

## Results and Discussion

### Acidic-acidic residue pairs in ASIC1

In the ASIC1 crystal structure, there exist 4 pairs of unusually close acidic-acidic residues: (a) Asp238-Asp350, (b) Glu220-Asp408, (c) Glu239-Asp346, and (d) Glu80-Glu417 (Figure 1). Since the carboxyl O-O atom distances in these pairs range between 2.8 and 3.0 Å, it was proposed that at least 1 of the acidic residues in each pair should be protonated, and that they form the primary sites for pH sensing in ASIC1 [5]. Indeed, the calculated titration curves of these pairs of acidic residues showed unusually high protonation probability (compare, for instance, with the titration curves in Ref. [12]), indicating that each pair has at least 1 protonated acidic residue.

#### (a) Asp238-Asp350 and Glu220-Asp408 pairs

**Figure 2:**
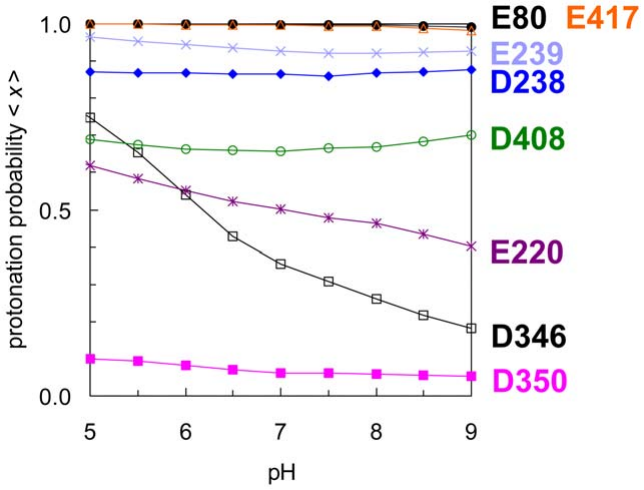
Protonation probabilities <*x*> [21] of the acidic-acidic residue pairs in ASIC1.

In the Asp238-Asp350 pair, Asp238 was mostly protonated and Asp350 was completely deprotonated at all the pH investigated (pH 5–9) (Figure 2). In the Glu220-Asp408 pair, although Glu220 was considerably protonated, Asp408 was more protonated than Glu220. It appears from Figure 2 that this pair of acidic residues binds a total of 1–1.5 H^+^ at pH 5–9.

#### (b) Glu239-Asp346 pair

In the Glu239-Asp346 pair, Glu239 was permanently protonated at pH 5–9 (Figure 2). The Glu239-Asp346 pair is located at the interface between the finger and the thumb domains of the ASIC1 protein, considerably away from the conducting pore of the transmembrane domain (Figure 1). Nevertheless, the mutation of Asp346 to Asn resulted in a significant shift in the pH_50_ value (i.e., pH of half-maximal activation) on the pH-dose-response curve; thus, Asp346 was proposed to play a key role in the pH-sensing mechanism of ASIC1 [5].

The pH_50_ value on the pH-dose-response curve of the wild-type ASIC1 protein has been experimentally determined to be 6.7 [5]. Interestingly, in the present study, among all the acidic-acidic residue pairs, Asp346 was the only residue with an apparent p*K*_a_ obtained from the pH-dependent titration curve (Figure 2) of ≈6–6.5. Furthermore, the p*K*_a_ of Asp346 obtained from protonation energy at pH 7 (see the method section for definition) was also 6.7 (subunit A in Table 1), and this value was in agreement with the experimentally measured pH_50_ value of 6.7 [5]. Thus, assuming that the pH-dependent domain movement plays a key role in pH sensing, Asp346 can be most likely the pH-sensing site of ASIC1 in terms of its remarkable p*K*_a_ value.

**Table 1:** The p*K*_a_ of 4 acidic-acidic residue pairs obtained from protonation energy at pH 7 (see Materials and Methods for the definition).

	residue	p*K*_a_			residue	p*K*_a_		
Subunit		A	B	C		A	B	C
	Asp238	7.8	7.6	7.4	Asp350	5.8	6.2	6.5
	Glu220	7.0	**8.9**	7.2	Asp408	7.3	**11.5**	7.1
	Glu239	8.1	8.1	**9.7**	Asp346	6.7	7.0	7.3
	Glu80	9.7	9.8	9.6	Glu417	9.5	9.2	9.6

Asp238-Asp350, Glu239-Asp346, Glu220-Asp408, and Glu80-Glu417 in ASIC1 (p*K*_a_). These p*K*_a_ values were obtained by calculating the protonation energy required to protonate the residue by 0.5 H^+^ at pH 7. Therefore, the values may differ from the apparent p*K*_a_ values obtained from the titration curves (see the method section for the definition) in Figure 1.

#### (c) Glu80-Glu417 pair

**Figure 3:**
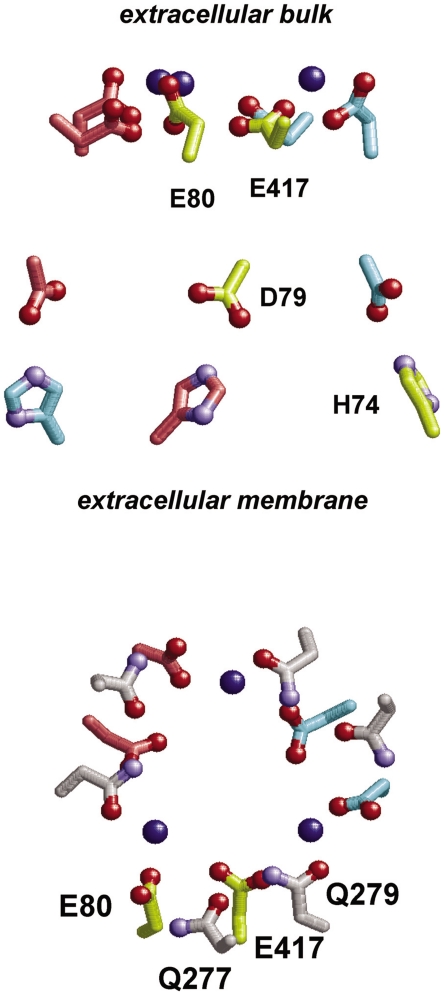
An acidic-residue ring that comprises Glu80 and Glu417. (Left) side view and (right) top view from the extracellular bulk. Carbon atoms of the residues that belong to the same subunit are depicted in the same color (yellow, pink, and cyan), while those of Gln277 and Gln279 are in white in all the subunits. Gln277 and Gln279 are depicted as sticks. The water oxygen atoms at Glu80 are depicted as blue spheres.

Notably, both Glu80 and Glu417 were completely protonated at pH 5–9, implying that this pair possesses ≈2 H^+^ (Figure 2). Three pairs of Glu80-Glu417 were located on the inner pore of the ASIC1 trimer, and they formed a ring comprising 6 acidic residues (Figure 3). Although all crystal waters were absent during the computations (see Materials and Methods for discussion), this completely protonated state of the Glu80-Glu417 pair can be anticipated based on the existence of a water molecule in the ASIC1 crystal structure at an H-bonding distance from the carboxyl oxygen atom of Glu80 (O_Glu80_-O_water_ distance = 2.8 Å). Without this water molecule, the positioning of these acidic residues at very small distances (Figure 3) would be unusually energetically unstable to exist in the crystal structure. The following 2 factors may be considered responsible for this unusually high protonation state of the acidic-acidic ring: (i) the specific arrangement of the acidic-acidic residues interacting ring that comprises 6 acidic residues, and (ii) the unfeasibility of solvation in the inner pore where these acidic residues are located.

The 6 acidic residues that form a ring in the pore may be considered to significantly favor protonation of each acidic residue because each residue in the ring has 2 acidic residues on either side (Figure 3, right). To investigate whether this arrangement is a primary factor for the unusually high protonation state of the Glu80-Glu417 pair, only the 6-residue ring (3 Glu80-Glu417 pairs) was isolated from the ASIC1 protein, solvated the acidic ring into an aqueous solution (represented by the dielectric constant *ε_w_* = 80), and titrated it at pH 5–9. From the titration curves (File S1), the apparent p*K*_a_ values of Glu80 and Glu417 were determined to be ≈5.3 and ≈6–6.3, respectively. Although these p*K*_a_ values are slightly higher than the standard p*K*_a_ value for Glu in an aqueous solution (4.4), the present study revealed that the isolated Glu80-Glu417 ring itself is not highly protonated in an aqueous solution at pH 7. It is noteworthy that the protonation states of the isolated Glu80-Glu417 ring in an aqueous solution remained unchanged when their H-bonding partners were added, namely, Gln277 and Gln279 (data not shown).

Further, the protonation states of the 6-residue ring in the uncharged ASIC1 protein were also investigated where all the atomic charges, except those of the Glu80-Glu417 pairs, were set to 0. In this uncharged protein environment, the 6 acidic residues were found to be completely protonated at all pH investigated (pH 5–9; data not shown). In the uncharged ASIC1, although the influence of the atomic charges of the proteins is absent, the protein volume still prevents solvation of the 6 titratable acidic residues.

From this result, it can be concluded that the loss of solvation in the ASIC1 protein environment is a factor required to explain the unusually high protonation state of the acidic-acidic ring in ASIC1. In general, in the inner pore of channel proteins, the loss of solvation (rather than repulsive interactions) is the major contributor toward destabilizing the charged groups [9], [13]. While the ASIC1 pore is formed by a number of charged and polar residues, it appears that bulk water access is limited there, similar to other ion-channel proteins. It can also be concluded that the unfeasibility of solvation is a primary factor for the unionized, highly protonated states of the Glu80-Glu417 pairs.

Glu80 is conserved in all the pH-sensitive ASICs. Mutations of the corresponding residue in ASIC3 enhance the rate of channel inactivation [8], while those of the residues in ASIC2a render the channels proton insensitive [7]. Thus, the highly protonated Glu80 demonstrated in the present study on ASIC1 may imply its possible role as a proton-binding site in ASICs.

### Asymmetry of the subunits A, B, and C in the ASIC1 trimer form

Although ASIC1 comprises 3 homo-trimer subunits A, B, and C, the ASIC1 crystal structure shows marked differences among their subunit conformations. In particular, different conformations of the transmembrane domains among the 3 subunits have been reported (see Figure 2b in Ref. [5]). It is debatable whether the structural asymmetry is inherent to the trimer [5]. In the present study, the generation of atomic coordinates of H atoms led to different H-bonding networks among these residues. Consequentially, different protonation states of the Glu220-Asp408 and Glu239-Asp346 pairs among the 3 subunits were observed (Table 1).

#### (a) Glu220-Asp408 pair

**Figure 4:**
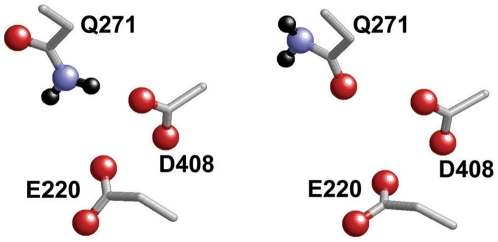
Orientation of the Gln271 side chain with respect to the Glu220-Asp408 residue pair. H atoms are depicted as black spheres.

The p*K*_a_ values obtained from protonation energy at pH 7 of both the residues in the Glu220-Asp408 pair of subunit B are higher by ≈2–4 than those of subunits A and C (Table 1). This is due to the proximity of the side chain O atom of Gln271 to the carboxyl O atom of Asp408 in subunit B (O-O distance = 3.0 Å) in the ASIC1 crystal structure (Figure 4, right). In contrast, the side chain N atom of Gln271 forms an H bond with the carboxyl O atom of Asp408 in subunits A and C, stabilizing the ionized state of Asp408 (Figure 4, left) and thus lowering its p*K*_a_ value. Although the different conformation of the Gln271 side chain is a potentially interesting finding, currently, it cannot be conclusively determined whether this is functionally relevant because of the limited resolution of 1.9 Å [5] in the ASIC1 crystal structure at which N and O atoms are unlikely to be distinctly distinguished.

#### (b) Glu239-Asp346 pair

**Figure 5:**
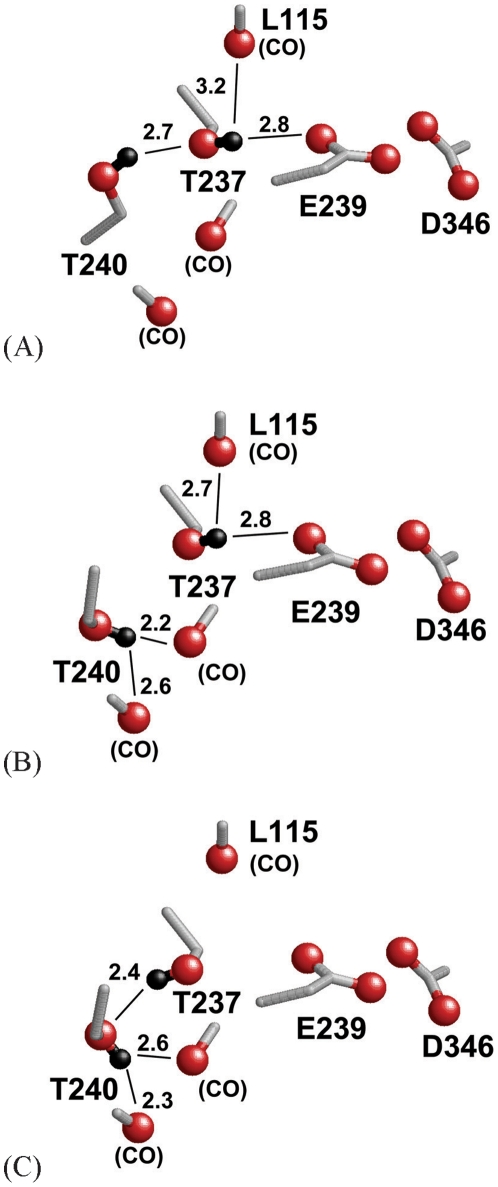
Discrepancy of the H-bonding network that involves the Glu239-Asp346 pair. (A) subunit A, (B) subunit B, and (C) subunit C. The H atoms are depicted as black spheres. Solid lines indicate the orientation of the H bond, and the numbers indicate the length between the H atom and the H-bond donor O atom in Å.

In the Glu239-Asp346 pair, the p*K*_a_ value obtained from protonation energy at pH 7 of Glu239 of subunit C was higher by 2 than the corresponding values of subunits A and B (Table 1). Therefore, a significantly different H-bond pattern involving mainly Glu239, Thr237, and Thr240 was found. The hydroxyl group of Thr237 formed a weak H bond with 1 of the carboxyl O atoms of Glu239 in subunits A and B (Figures 5A and 5B, respectively), while the corresponding H bond was absent in subunit C (O_Thr237_–O_Glu239_ distance = 3.7–3.8 Å; Figure 5C). Thus, the ionized state of Glu239 in subunits A and B can be stabilized, leading to the downshift in the p*K*_a_ value of Glu239 as compared to that in subunit C.

Furthermore, there was another significant difference in protein conformations among the subunits: not only the H-bond pattern but also the orientation of Thr240 differed significantly between subunit A and subunits B and C. In subunit A, the Thr240 side chain was oriented toward the hydroxyl O atom of Thr237, forming an H bond (O_Thr240_–O_Thr237_ distance = 3.5 Å; Figure 5A). However, the H bond between the Thr240 and Thr237 side chains observed in subunit A was absent in subunit B, because the hydroxyl O atom of Thr240 is considerably closer to the carbonyl O atoms of the protein backbone at Thr237 and Thr240 (O_Thr240(OH)_–O_Thr237(CO)_ distance = 3.1 Å, O_Thr240(OH)_–O_Thr240(CO)_ distance = 3.1 Å) than to the hydroxyl O atom of Thr237 (O_Thr240(OH)_–O_Thr237(CO)_ distance = 3.7 Å). Thus, the Thr240 side chain, in turn, formed an H bond with the carbonyl O atoms of the protein backbone at Thr237 and Thr240 (Figure 5B). In subunit C, Thr237 did not form an H bond with Glu239 but formed an H bond with Thr240, because the donor-acceptor distance of the H bond is lesser in the latter (O_Thr240_–O_Thr237_ distance = 3.3 Å) than in the former (O_Thr237_–O_Glu239_ distance = 3.8 Å). Therefore, the ionized state of Glu237 is less stable in subunit C than in subunits A and B because of the lack of the H bond from Thr237 to Glu237 (Figure 5C), leading to the upshifting of its p*K*_a_ by 1.6 (Table 1). In contrast to the significant difference in the p*K*_a_ of Glu239 (which was affected by the H-bond pattern of Thr237), the p*K*_a_ of the pair-partner residue Asp346 did not vary significantly (Table 1). This was due to the identical protonation state of its pair-partner residue Glu239 (i.e., Asp346 was essentially protonated in each subunit and its protonation state did not alter among the subunits).

Of the residues that participate in the H-bonding network of the Glu239-Asp346 pair (i.e., Glu239, Asp346, Thr237, and Thr240), Glu239 and Thr240 are highly conserved residues in ASICs. On the other hand, Asp346 and Thr237 are highly conserved in all ASICs but ASIC3 (see supplementary information in Ref. [5]). In ASIC3, Asp346 and Thr237 are replaced with Ser and Met (rat and mouse) or His and Asn (human), respectively. The fact that Asp346 and Thr237 are simultaneously replaced in ASIC3 implies that these 2 residues probably function cooperatively in ASICs, as indicated in the highly associated H-bonding network shown in Figure 5. Assuming that Asp346 plays a key role in the proposed pH-sensing mechanism of ASIC1 [5], Thr237 may also cooperate with Asp346 in ASIC1 pH sensing. In addition, the absence of the residues corresponding to Asp346 and Thr237 of ASIC1 in ASIC3 suggests that the proposed pH-sensing role of Asp346 in ASIC1 [5] does not hold true for the pH-sensing mechanism of ASIC3 (the pH-sensing mechanism has been further described later in the Discussion).

The only ASIC1 crystal structure currently available was obtained at a low pH and has been proposed to represent a thermodynamically favorable desensitized state [5]. The desensitized state can transform into the open state and the closed state [14]. The different H-bonding pattern and protein conformations of residues Glu239, Asp346, Thr237, and Thr240 of each subunit revealed in the present study may imply possible variations of the ASIC1 conformations in other channel states (i.e., including the open and closed states).

### Residues that possess p*K*_a_ near pH_50_ = 6.7

Since the pH_50_ value on the pH-dose-response curve of the wild-type ASIC1 protein is 6.7 [5], it is worthwhile to reveal residues that possess p*K*_a_ value of ≈6.7 for understanding the pH-sensing mechanism of ASIC1. Among the acidic residues that are not involved in the acidic-acidic residue pairs, only Glu299 and Glu343 (apparent p*K*_a_ of ≈7 and 6 obtained from the titration curves, respectively) showed their p*K*_a_ values at similar levels (File S1). However, since these residues are located on the protein surface, they are probably not likely to be involved in the pH-sensing mechanism of ASIC1.

Among the acidic-acidic residue pairs, only Asp346 was found to possess p*K*_a_ within this range in the present study (Table 1). Although Asp346 is highly conserved among ASICs, it is replaced with a nontitratable residue (Asn) in ASIC1 from lamprey. Interestingly, ASIC1 from lamprey, which lacks Asp346, is proton insensitive, which is in contrast to the proton-sensitive ASIC1 from chicken [15]; this fact may support the proposed role of Asp346 in the pH sensing mechanism of ASIC1 from chicken [5]. However, it should be noted that Asp346 is not conserved even in the proton-sensitive ASIC3. Therefore, it might be speculated that either (i) ASIC3 has a pH-sensing mechanism different from that of ASIC1 or (ii) both ASIC1 and ASIC3 have another common residue(s) that also plays a pH-sensing role.

**Figure 6:**
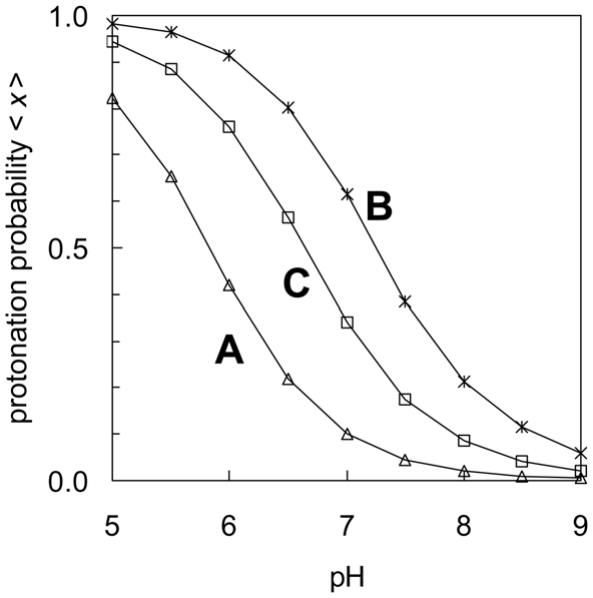
Protonation probabilities <*x*> of His74 in subunits A, B, and C.

Regarding non-acidic residues, His74 (Figure 6, subunit C) and His111 (File S1) were found to possess an apparent p*K*_a_ of ≈6–7 in the present study, although the latter was located on the protein surface. It is noteworthy that no other His residues have their p*K*_a_ values at this range of pH, irrespective of the fact that the p*K*_a_ of an isolated His residue is generally ≈7 (File S1). Mutation of a His residue corresponding to His74 to Ala in ASIC2a resulted in the deficiency of pH sensitivity [6], [7]. This His residue can also be observed in the proton-sensitive ASIC2a but not in the proton-insensitive ASIC2b. His74, therefore, has been proposed to be involved in the activation of ASIC2a by protons [7]. The ASIC1 crystal structure revealed that His74 is located on the inner cavity surface on the 3-fold axis (Figure 3) [5]. Thus, His74 is probably the residue that can directly tune the gating path in terms of the protonation states of the conducting pore near pH_50_. Interestingly, His74 is also conserved in the proton-sensitive ASIC3 that lacks Asp346. Since both ASIC1 and ASIC3 exhibit the highest proton affinity among the ASICs, the presence of a residue corresponding to His74 in ASIC3 may explain why ASIC3 is capable of sensing pH without possessing an acidic residue corresponding to Asp346.

## Materials and Methods

### Atomic coordinates and charges

For performing computations of the ASIC1 trimer form, the crystal structure of ASIC1, comprising subunits A, B, and C, from chicken at 1.9 Å resolution was used (protein data bank [PDB] code: 2QTS) [5]. The atomic coordinates were obtained using the same procedures used in previous studies on channel proteins [9], [10], [11], [12]. The positions of H atoms were energetically optimized with CHARMM [16] by using the CHARMM22 force field [17]. While carrying out this procedure, the positions of all non-H atoms were fixed, and the standard charge states of all the titratable groups were maintained, i.e., the basic and acidic groups were considered protonated and deprotonated, respectively. All the other atoms whose coordinates were available in the crystal structure were not geometrically optimized. Atomic partial charges of the amino acids were adopted from the all-atom CHARMM22 parameter set [16].

### Protonation pattern

The present computation is based on the electrostatic continuum model created by solving the LPB equation with the MEAD program [18]. To facilitate a direct comparison with previous computational results, identical computational conditions and parameters such as atomic partial charges and dielectric constants were used (e.g., Refs. [9], [10], [11], [12], [19]). The ensemble of the protonation patterns was sampled using the Monte Carlo (MC) method with the Karlsberg program (Rabenstein, B. *Karlsberg online manual*, http://agknapp.chemie.fu-berlin.de/karlsberg/). The dielectric constant was set to *ε_p_* = 4 inside the protein and to *ε_w_* = 80 for solvent and protein cavities corresponding to water. All computations were performed at 300 K, pH 5–9, and an ionic strength of 100 mM. The LPB equation was solved using a 3-step grid-focusing procedure with a starting grid resolution of 2.5 Å, an intermediate grid resolution of 1.0 Å, and a final grid resolution of 0.3 Å. MC sampling yields the probabilities [*A* ^−^] and [*AH*] of the deprotonated and protonated states of the titratable residue *A*, respectively.

### Dielectric volume

As a general and uniform strategy, all crystal waters were removed during the computations (for instance, Refs. [9], [10], [11], [12], [19]) due to the lack of experimental information on hydrogen atom positions. Cavities resulting from the removal of crystal waters were uniformly filled with a solvent dielectric medium of *ε* = 80. Thus, effectively, the effect of the removed water molecules was compensated for implicitly by the high value of the dielectric constant in these cavities. A discussion on the appropriate value of the dielectric constant in proteins for electrostatic energy computations can be found in Ref. [20].

### Definition of p*K*_a_

#### a) Apparent pK_a_ obtained from the titration curve

Protonation probability <*x*> is defined as <*x*> = [*AH*]/([*AH*]+[*A* ^−^]) [21]. Once the residue titration curve (<x> versus pH) is obtained by changing the pH of bulk aqueous solution and then plotting <*x*> of the residue, the p*K*_a_ value can be obtained as the pH at the point where <*x*> = 0.5 on the titration curve. In this manuscript, this p*K*_a_ is called “*apparent pK*_a_ *obtained from the titration curve*.”

#### b) pK_a_ obtained from protonation energy at pH 7

However, titration of acidic-acidic pairs often yields a titration curve that never decreased to <*x*> = 0.5 at the investigated pH. Even if the titration curve reaches <*x*> = 0.5, the slope at <*x*> = 0.5 may be too gentle to determine a unique *apparent pK*_a_ value appropriately. In such a case, one could use an alternative value of p*K*_a_ that can be obtained by calculating the energy required to yield 0.5 H^+^ protonation (i.e., <*x*> = 0.5) of the residue at pH 7 (*pK*_a_ *obtained from protonation energy at pH 7*). When determining this p*K*_a_ value, the focusing residue possesses <*x*> = 0.5, while all the other titratable sites possess protonation states equilibrated at pH 7. This p*K*_a_ value can always be obtained uniquely at a specific pH of bulk water.

Since only a single residue of the protein cannot be experimentally titrated, in general, the apparent p*K*_a_ obtained from the titration curve rather than the p*K*_a_ obtained from protonation energy is considered to correspond to an experimentally determined p*K*_a_. The discrepancy between the p*K*_a_ values defined above cannot be ignored, particularly when the focusing site is subjected to an unusually large influence of the surrounding charged residues (e.g., close positioning of the acidic-acidic residue pair). Nevertheless, in cases wherein such a strong charge influence from the surrounding residues can be ignored, the p*K*_a_ obtained from protonation energy at pH 7 is often essentially identical to the apparent p*K*_a_ obtained from the titration curve and provides relevant information of the protonation state (see, for instance, Ref. [12]).

In the present study, using the Henderson-Hasselbalch equation, p*K*_a_ obtained from protonation energy at pH 7 was calculated as the formal pH at which the concentrations of [*A* ^−^] and [*AH*] are equal. The procedures to obtain p*K*_a_ of the titratable residues are identical to those used to determine the redox potential for redox-active groups; the Nernst equation is applied in the latter case [21]. Therefore, the accuracy of the present p*K*_a_ computations is directly comparable to that of former computations of redox-active cofactors (e.g., [22], [23]). From this analogy, the numerical error of p*K*_a_ computation can be estimated to be ≈0.2 pH units. Systematic errors, which typically relate to specific conformations that may differ from the given crystal structures, can be considerably larger sometimes. Since the calculations in present study were performed under the same conditions as our previous p*K*_a_ computation for other channel proteins, further details on error estimates and comparisons with the previous results can be obtained in Refs. [9], [10], [11], [12].

## Supporting Information

File S1 **Protonation probabilities of titratable residues.**
(DOC)
