# Supplementary material for: Proton-Binding Sites of Acid-Sensing Ion Channel 1
Source: PLoS One. 2011 Feb 14;6(2):e16920. doi: 10.1371/journal.pone.0016920 (PMC3038902; doi:10.1371/journal.pone.0016920)
Supplement: File S1 — Protonation probabilities of titratable residues. (DOC) [file pone.0016920.s001.doc]

**Supporting Information S1**

Proton-binding sites of acid-sensing ion channel 1

Hiroshi Ishikita

E417 (water)

E80 (water)

Figure S1. Protonation probabilities <*x*> of the Glu80 (black lines) - Glu417 (red lines) pair solvated in an aqueous solution. Titration curves of all the Glu80-Glu417 pairs in subunits A, B, and C are shown without being distinguished.

Table S1. Protonation probabilities of titratable residues in the ASIC1 crystal structure. Values 0 and 1 correspond to fully deprotonated and fully protonated states of the residues, respectively.

| **pH** |  |  |  | **5** | **5.5** | **6** | **6.5** | **7** | **7.5** | **8** | **8.5** | **9** |
| --- | --- | --- | --- | --- | --- | --- | --- | --- | --- | --- | --- | --- |
|  | subunit A | **ARG** | **44** | 1.00 | 1.00 | 1.00 | 1.00 | 1.00 | 1.00 | 1.00 | 1.00 | 1.00 |
|  |  | **ARG** | **65** | 1.00 | 1.00 | 1.00 | 1.00 | 1.00 | 1.00 | 1.00 | 1.00 | 1.00 |
|  |  | **ARG** | **85** | 1.00 | 1.00 | 1.00 | 1.00 | 1.00 | 1.00 | 1.00 | 1.00 | 1.00 |
|  |  | **ARG** | **100** | 1.00 | 1.00 | 1.00 | 1.00 | 1.00 | 1.00 | 1.00 | 1.00 | 1.00 |
|  |  | **ARG** | **103** | 1.00 | 1.00 | 1.00 | 1.00 | 1.00 | 1.00 | 1.00 | 1.00 | 1.00 |
|  |  | **ARG** | **122** | 1.00 | 1.00 | 1.00 | 1.00 | 1.00 | 1.00 | 1.00 | 1.00 | 1.00 |
|  |  | **ARG** | **146** | 1.00 | 1.00 | 1.00 | 1.00 | 1.00 | 1.00 | 1.00 | 1.00 | 1.00 |
|  |  | **ARG** | **161** | 1.00 | 1.00 | 1.00 | 1.00 | 1.00 | 1.00 | 1.00 | 1.00 | 1.00 |
|  |  | **ARG** | **167** | 1.00 | 1.00 | 1.00 | 1.00 | 1.00 | 1.00 | 1.00 | 1.00 | 1.00 |
|  |  | **ARG** | **176** | 1.00 | 1.00 | 0.99 | 0.98 | 0.96 | 0.92 | 0.83 | 0.71 | 0.53 |
|  |  | **ARG** | **191** | 1.00 | 1.00 | 1.00 | 1.00 | 1.00 | 1.00 | 1.00 | 1.00 | 1.00 |
|  |  | **ARG** | **207** | 1.00 | 1.00 | 1.00 | 1.00 | 1.00 | 1.00 | 1.00 | 1.00 | 1.00 |
|  |  | **ARG** | **280** | 1.00 | 1.00 | 1.00 | 1.00 | 1.00 | 1.00 | 1.00 | 1.00 | 1.00 |
|  |  | **ARG** | **310** | 1.00 | 1.00 | 1.00 | 1.00 | 1.00 | 1.00 | 0.99 | 0.99 | 0.98 |
|  |  | **ARG** | **316** | 1.00 | 1.00 | 1.00 | 1.00 | 1.00 | 1.00 | 1.00 | 1.00 | 1.00 |
|  |  | **ARG** | **325** | 1.00 | 1.00 | 1.00 | 1.00 | 1.00 | 1.00 | 1.00 | 1.00 | 1.00 |
|  |  | **ARG** | **370** | 1.00 | 1.00 | 1.00 | 1.00 | 1.00 | 1.00 | 1.00 | 1.00 | 1.00 |
|  | **subunit B** | **ARG** | **44** | 1.00 | 1.00 | 1.00 | 1.00 | 1.00 | 1.00 | 1.00 | 1.00 | 1.00 |
|  |  | **ARG** | **65** | 1.00 | 1.00 | 1.00 | 1.00 | 1.00 | 1.00 | 1.00 | 1.00 | 1.00 |
|  |  | **ARG** | **85** | 1.00 | 1.00 | 1.00 | 1.00 | 1.00 | 1.00 | 1.00 | 1.00 | 1.00 |
|  |  | **ARG** | **100** | 1.00 | 1.00 | 1.00 | 1.00 | 1.00 | 1.00 | 1.00 | 1.00 | 1.00 |
|  |  | **ARG** | **103** | 1.00 | 1.00 | 1.00 | 1.00 | 1.00 | 1.00 | 1.00 | 1.00 | 1.00 |
|  |  | **ARG** | **122** | 1.00 | 1.00 | 1.00 | 1.00 | 1.00 | 1.00 | 1.00 | 1.00 | 1.00 |
|  |  | **ARG** | **146** | 1.00 | 1.00 | 1.00 | 1.00 | 1.00 | 1.00 | 1.00 | 1.00 | 1.00 |
|  |  | **ARG** | **161** | 1.00 | 1.00 | 1.00 | 1.00 | 1.00 | 1.00 | 1.00 | 1.00 | 1.00 |
|  |  | **ARG** | **167** | 1.00 | 1.00 | 1.00 | 1.00 | 1.00 | 1.00 | 1.00 | 1.00 | 1.00 |
|  |  | **ARG** | **176** | 1.00 | 1.00 | 1.00 | 1.00 | 1.00 | 1.00 | 1.00 | 1.00 | 1.00 |
|  |  | **ARG** | **191** | 1.00 | 1.00 | 1.00 | 1.00 | 1.00 | 1.00 | 1.00 | 1.00 | 1.00 |
|  |  | **ARG** | **207** | 1.00 | 1.00 | 1.00 | 1.00 | 1.00 | 1.00 | 1.00 | 1.00 | 1.00 |
|  |  | **ARG** | **280** | 1.00 | 1.00 | 1.00 | 1.00 | 1.00 | 1.00 | 1.00 | 1.00 | 1.00 |
|  |  | **ARG** | **310** | 0.99 | 0.99 | 0.99 | 0.99 | 0.98 | 0.97 | 0.97 | 0.96 | 0.96 |
|  |  | **ARG** | **316** | 1.00 | 1.00 | 1.00 | 1.00 | 1.00 | 1.00 | 1.00 | 1.00 | 1.00 |
|  |  | **ARG** | **325** | 1.00 | 1.00 | 1.00 | 1.00 | 1.00 | 1.00 | 1.00 | 1.00 | 1.00 |
|  |  | **ARG** | **370** | 1.00 | 1.00 | 1.00 | 1.00 | 1.00 | 1.00 | 1.00 | 1.00 | 1.00 |
|  | **subunit C** | **ARG** | **44** | 1.00 | 1.00 | 1.00 | 1.00 | 1.00 | 1.00 | 1.00 | 1.00 | 1.00 |
|  |  | **ARG** | **65** | 1.00 | 1.00 | 1.00 | 1.00 | 1.00 | 1.00 | 1.00 | 1.00 | 1.00 |
|  |  | **ARG** | **85** | 1.00 | 1.00 | 1.00 | 1.00 | 1.00 | 1.00 | 1.00 | 1.00 | 1.00 |
|  |  | **ARG** | **100** | 1.00 | 1.00 | 1.00 | 1.00 | 1.00 | 1.00 | 1.00 | 1.00 | 1.00 |
|  |  | **ARG** | **103** | 1.00 | 1.00 | 1.00 | 1.00 | 1.00 | 1.00 | 1.00 | 1.00 | 1.00 |
|  |  | **ARG** | **122** | 1.00 | 1.00 | 1.00 | 1.00 | 1.00 | 1.00 | 1.00 | 1.00 | 1.00 |
|  |  | **ARG** | **146** | 1.00 | 1.00 | 1.00 | 1.00 | 1.00 | 1.00 | 1.00 | 1.00 | 1.00 |
|  |  | **ARG** | **161** | 1.00 | 1.00 | 1.00 | 1.00 | 1.00 | 1.00 | 1.00 | 1.00 | 1.00 |
|  |  | **ARG** | **167** | 1.00 | 1.00 | 1.00 | 1.00 | 1.00 | 1.00 | 1.00 | 1.00 | 1.00 |
|  |  | **ARG** | **176** | 1.00 | 1.00 | 1.00 | 1.00 | 1.00 | 1.00 | 1.00 | 1.00 | 1.00 |
|  |  | **ARG** | **191** | 1.00 | 1.00 | 1.00 | 1.00 | 1.00 | 1.00 | 1.00 | 1.00 | 1.00 |
|  |  | **ARG** | **207** | 1.00 | 1.00 | 1.00 | 1.00 | 1.00 | 1.00 | 1.00 | 1.00 | 1.00 |
|  |  | **ARG** | **280** | 1.00 | 1.00 | 1.00 | 1.00 | 1.00 | 1.00 | 1.00 | 1.00 | 1.00 |
|  |  | **ARG** | **310** | 1.00 | 1.00 | 1.00 | 1.00 | 1.00 | 0.99 | 0.99 | 0.99 | 0.99 |
|  |  | **ARG** | **316** | 1.00 | 1.00 | 1.00 | 1.00 | 1.00 | 1.00 | 1.00 | 1.00 | 1.00 |
|  |  | **ARG** | **325** | 1.00 | 1.00 | 1.00 | 1.00 | 1.00 | 1.00 | 1.00 | 1.00 | 1.00 |
|  |  | **ARG** | **370** | 1.00 | 1.00 | 1.00 | 1.00 | 1.00 | 1.00 | 1.00 | 1.00 | 1.00 |
|  | **subunit A** | **ASP** | **79** | 0.00 | 0.00 | 0.00 | 0.00 | 0.00 | 0.00 | 0.00 | 0.00 | 0.00 |
|  |  | **ASP** | **108** | 0.00 | 0.00 | 0.00 | 0.00 | 0.00 | 0.00 | 0.00 | 0.00 | 0.00 |
|  |  | **ASP** | **127** | 0.06 | 0.02 | 0.01 | 0.00 | 0.00 | 0.00 | 0.00 | 0.00 | 0.00 |
|  |  | **ASP** | **132** | 0.14 | 0.09 | 0.04 | 0.02 | 0.01 | 0.00 | 0.00 | 0.00 | 0.00 |
|  |  | **ASP** | **141** | 0.26 | 0.14 | 0.06 | 0.02 | 0.01 | 0.00 | 0.00 | 0.00 | 0.00 |
|  |  | **ASP** | **160** | 0.00 | 0.00 | 0.00 | 0.00 | 0.00 | 0.00 | 0.00 | 0.00 | 0.00 |
|  |  | **ASP** | **165** | 0.00 | 0.00 | 0.00 | 0.00 | 0.00 | 0.00 | 0.00 | 0.00 | 0.00 |
|  |  | **ASP** | **184** | 0.00 | 0.00 | 0.00 | 0.00 | 0.00 | 0.00 | 0.00 | 0.00 | 0.00 |
|  |  | **ASP** | **203** | 0.08 | 0.03 | 0.01 | 0.00 | 0.00 | 0.00 | 0.00 | 0.00 | 0.00 |
|  |  | **ASP** | **224** | 0.00 | 0.00 | 0.00 | 0.00 | 0.00 | 0.00 | 0.00 | 0.00 | 0.00 |
|  |  | **ASP** | **228** | 0.30 | 0.17 | 0.09 | 0.04 | 0.02 | 0.01 | 0.01 | 0.00 | 0.00 |
|  |  | **ASP** | **238** | 0.87 | 0.87 | 0.87 | 0.87 | 0.86 | 0.86 | 0.87 | 0.87 | 0.88 |
|  |  | **ASP** | **254** | 0.02 | 0.01 | 0.00 | 0.00 | 0.00 | 0.00 | 0.00 | 0.00 | 0.00 |
|  |  | **ASP** | **260** | 0.00 | 0.00 | 0.00 | 0.00 | 0.00 | 0.00 | 0.00 | 0.00 | 0.00 |
|  |  | **ASP** | **290** | 0.16 | 0.08 | 0.04 | 0.01 | 0.01 | 0.00 | 0.00 | 0.00 | 0.00 |
|  |  | **ASP** | **297** | 0.17 | 0.07 | 0.03 | 0.01 | 0.00 | 0.00 | 0.00 | 0.00 | 0.00 |
|  |  | **ASP** | **302** | 0.15 | 0.06 | 0.02 | 0.01 | 0.00 | 0.00 | 0.00 | 0.00 | 0.00 |
|  |  | **ASP** | **312** | 0.01 | 0.01 | 0.01 | 0.01 | 0.00 | 0.00 | 0.00 | 0.00 | 0.00 |
|  |  | **ASP** | **332** | 0.05 | 0.02 | 0.01 | 0.00 | 0.00 | 0.00 | 0.00 | 0.00 | 0.00 |
|  |  | **ASP** | **346** | 0.75 | 0.65 | 0.54 | 0.43 | 0.35 | 0.31 | 0.26 | 0.22 | 0.18 |
|  |  | **ASP** | **350** | 0.10 | 0.09 | 0.08 | 0.07 | 0.06 | 0.06 | 0.06 | 0.05 | 0.05 |
|  |  | **ASP** | **356** | 0.00 | 0.00 | 0.00 | 0.00 | 0.00 | 0.00 | 0.00 | 0.00 | 0.00 |
|  |  | **ASP** | **408** | 0.69 | 0.67 | 0.66 | 0.66 | 0.66 | 0.66 | 0.67 | 0.68 | 0.70 |
|  |  | **ASP** | **433** | 0.00 | 0.00 | 0.00 | 0.00 | 0.00 | 0.00 | 0.00 | 0.00 | 0.00 |
|  |  | **ASP** | **454** | 0.09 | 0.04 | 0.02 | 0.01 | 0.00 | 0.00 | 0.00 | 0.00 | 0.00 |
|  | **subunit B** | **ASP** | **79** | 0.00 | 0.00 | 0.00 | 0.00 | 0.00 | 0.00 | 0.00 | 0.00 | 0.00 |
|  |  | **ASP** | **108** | 0.00 | 0.00 | 0.00 | 0.00 | 0.00 | 0.00 | 0.00 | 0.00 | 0.00 |
|  |  | **ASP** | **127** | 0.02 | 0.01 | 0.00 | 0.00 | 0.00 | 0.00 | 0.00 | 0.00 | 0.00 |
|  |  | **ASP** | **132** | 0.00 | 0.00 | 0.00 | 0.00 | 0.00 | 0.00 | 0.00 | 0.00 | 0.00 |
|  |  | **ASP** | **141** | 0.01 | 0.00 | 0.00 | 0.00 | 0.00 | 0.00 | 0.00 | 0.00 | 0.00 |
|  |  | **ASP** | **160** | 0.00 | 0.00 | 0.00 | 0.00 | 0.00 | 0.00 | 0.00 | 0.00 | 0.00 |
|  |  | **ASP** | **165** | 0.00 | 0.00 | 0.00 | 0.00 | 0.00 | 0.00 | 0.00 | 0.00 | 0.00 |
|  |  | **ASP** | **184** | 0.00 | 0.00 | 0.00 | 0.00 | 0.00 | 0.00 | 0.00 | 0.00 | 0.00 |
|  |  | **ASP** | **203** | 0.03 | 0.01 | 0.00 | 0.00 | 0.00 | 0.00 | 0.00 | 0.00 | 0.00 |
|  |  | **ASP** | **224** | 0.00 | 0.00 | 0.00 | 0.00 | 0.00 | 0.00 | 0.00 | 0.00 | 0.00 |
|  |  | **ASP** | **228** | 0.48 | 0.27 | 0.14 | 0.07 | 0.03 | 0.02 | 0.01 | 0.01 | 0.00 |
|  |  | **ASP** | **238** | 0.85 | 0.84 | 0.82 | 0.81 | 0.81 | 0.82 | 0.83 | 0.85 | 0.86 |
|  |  | **ASP** | **254** | 0.00 | 0.00 | 0.00 | 0.00 | 0.00 | 0.00 | 0.00 | 0.00 | 0.00 |
|  |  | **ASP** | **260** | 0.00 | 0.00 | 0.00 | 0.00 | 0.00 | 0.00 | 0.00 | 0.00 | 0.00 |
|  |  | **ASP** | **290** | 0.09 | 0.04 | 0.01 | 0.01 | 0.00 | 0.00 | 0.00 | 0.00 | 0.00 |
|  |  | **ASP** | **297** | 0.11 | 0.04 | 0.01 | 0.01 | 0.00 | 0.00 | 0.00 | 0.00 | 0.00 |
|  |  | **ASP** | **302** | 0.05 | 0.02 | 0.01 | 0.00 | 0.00 | 0.00 | 0.00 | 0.00 | 0.00 |
|  |  | **ASP** | **312** | 0.91 | 0.88 | 0.86 | 0.86 | 0.85 | 0.86 | 0.82 | 0.72 | 0.53 |
|  |  | **ASP** | **332** | 0.05 | 0.02 | 0.01 | 0.00 | 0.00 | 0.00 | 0.00 | 0.00 | 0.00 |
|  |  | **ASP** | **346** | 0.82 | 0.76 | 0.66 | 0.57 | 0.49 | 0.41 | 0.34 | 0.28 | 0.23 |
|  |  | **ASP** | **350** | 0.14 | 0.13 | 0.14 | 0.14 | 0.13 | 0.11 | 0.10 | 0.08 | 0.07 |
|  |  | **ASP** | **356** | 0.00 | 0.00 | 0.00 | 0.00 | 0.00 | 0.00 | 0.00 | 0.00 | 0.00 |
|  |  | **ASP** | **408** | 1.00 | 1.00 | 1.00 | 1.00 | 1.00 | 1.00 | 1.00 | 1.00 | 1.00 |
|  |  | **ASP** | **433** | 0.00 | 0.00 | 0.00 | 0.00 | 0.00 | 0.00 | 0.00 | 0.00 | 0.00 |
|  |  | **ASP** | **454** | 0.94 | 0.88 | 0.80 | 0.67 | 0.47 | 0.26 | 0.12 | 0.05 | 0.02 |
|  | **subunit C** | **ASP** | **79** | 0.00 | 0.00 | 0.00 | 0.00 | 0.00 | 0.00 | 0.00 | 0.00 | 0.00 |
|  |  | **ASP** | **108** | 0.00 | 0.00 | 0.00 | 0.00 | 0.00 | 0.00 | 0.00 | 0.00 | 0.00 |
|  |  | **ASP** | **127** | 0.04 | 0.02 | 0.01 | 0.00 | 0.00 | 0.00 | 0.00 | 0.00 | 0.00 |
|  |  | **ASP** | **132** | 0.00 | 0.00 | 0.00 | 0.00 | 0.00 | 0.00 | 0.00 | 0.00 | 0.00 |
|  |  | **ASP** | **141** | 0.45 | 0.26 | 0.12 | 0.04 | 0.02 | 0.01 | 0.00 | 0.00 | 0.00 |
|  |  | **ASP** | **160** | 0.00 | 0.00 | 0.00 | 0.00 | 0.00 | 0.00 | 0.00 | 0.00 | 0.00 |
|  |  | **ASP** | **165** | 0.00 | 0.00 | 0.00 | 0.00 | 0.00 | 0.00 | 0.00 | 0.00 | 0.00 |
|  |  | **ASP** | **184** | 0.01 | 0.01 | 0.00 | 0.00 | 0.00 | 0.00 | 0.00 | 0.00 | 0.00 |
|  |  | **ASP** | **203** | 0.03 | 0.01 | 0.00 | 0.00 | 0.00 | 0.00 | 0.00 | 0.00 | 0.00 |
|  |  | **ASP** | **224** | 0.00 | 0.00 | 0.00 | 0.00 | 0.00 | 0.00 | 0.00 | 0.00 | 0.00 |
|  |  | **ASP** | **228** | 0.54 | 0.36 | 0.22 | 0.13 | 0.08 | 0.04 | 0.03 | 0.01 | 0.01 |
|  |  | **ASP** | **238** | 0.83 | 0.82 | 0.79 | 0.75 | 0.71 | 0.69 | 0.71 | 0.74 | 0.78 |
|  |  | **ASP** | **254** | 0.00 | 0.00 | 0.00 | 0.00 | 0.00 | 0.00 | 0.00 | 0.00 | 0.00 |
|  |  | **ASP** | **260** | 0.00 | 0.00 | 0.00 | 0.00 | 0.00 | 0.00 | 0.00 | 0.00 | 0.00 |
|  |  | **ASP** | **290** | 0.28 | 0.16 | 0.08 | 0.03 | 0.01 | 0.01 | 0.00 | 0.00 | 0.00 |
|  |  | **ASP** | **297** | 0.13 | 0.06 | 0.02 | 0.01 | 0.00 | 0.00 | 0.00 | 0.00 | 0.00 |
|  |  | **ASP** | **302** | 0.16 | 0.06 | 0.02 | 0.01 | 0.00 | 0.00 | 0.00 | 0.00 | 0.00 |
|  |  | **ASP** | **312** | 0.01 | 0.01 | 0.01 | 0.01 | 0.00 | 0.00 | 0.00 | 0.00 | 0.00 |
|  |  | **ASP** | **332** | 0.06 | 0.02 | 0.01 | 0.00 | 0.00 | 0.00 | 0.00 | 0.00 | 0.00 |
|  |  | **ASP** | **346** | 0.95 | 0.91 | 0.86 | 0.78 | 0.69 | 0.61 | 0.51 | 0.40 | 0.28 |
|  |  | **ASP** | **350** | 0.17 | 0.17 | 0.19 | 0.21 | 0.23 | 0.23 | 0.21 | 0.18 | 0.13 |
|  |  | **ASP** | **356** | 0.00 | 0.00 | 0.00 | 0.00 | 0.00 | 0.00 | 0.00 | 0.00 | 0.00 |
|  |  | **ASP** | **408** | 0.66 | 0.61 | 0.58 | 0.56 | 0.56 | 0.57 | 0.59 | 0.59 | 0.60 |
|  |  | **ASP** | **433** | 0.00 | 0.00 | 0.00 | 0.00 | 0.00 | 0.00 | 0.00 | 0.00 | 0.00 |
|  |  | **ASP** | **454** | 0.00 | 0.00 | 0.00 | 0.00 | 0.00 | 0.00 | 0.00 | 0.00 | 0.00 |
|  | **subunit A** | **GLU** | **80** | 1.00 | 1.00 | 1.00 | 1.00 | 1.00 | 1.00 | 1.00 | 1.00 | 0.99 |
|  |  | **GLU** | **98** | 0.96 | 0.96 | 0.95 | 0.95 | 0.95 | 0.94 | 0.93 | 0.93 | 0.94 |
|  |  | **GLU** | **114** | 0.13 | 0.08 | 0.05 | 0.02 | 0.01 | 0.00 | 0.00 | 0.00 | 0.00 |
|  |  | **GLU** | **124** | 0.47 | 0.24 | 0.10 | 0.04 | 0.01 | 0.00 | 0.00 | 0.00 | 0.00 |
|  |  | **GLU** | **133** | 0.46 | 0.34 | 0.19 | 0.09 | 0.04 | 0.01 | 0.00 | 0.00 | 0.00 |
|  |  | **GLU** | **137** | 0.43 | 0.24 | 0.11 | 0.04 | 0.01 | 0.00 | 0.00 | 0.00 | 0.00 |
|  |  | **GLU** | **157** | 0.00 | 0.00 | 0.00 | 0.00 | 0.00 | 0.00 | 0.00 | 0.00 | 0.00 |
|  |  | **GLU** | **168** | 0.00 | 0.00 | 0.00 | 0.00 | 0.00 | 0.00 | 0.00 | 0.00 | 0.00 |
|  |  | **GLU** | **178** | 0.00 | 0.00 | 0.00 | 0.00 | 0.00 | 0.00 | 0.00 | 0.00 | 0.00 |
|  |  | **GLU** | **183** | 0.31 | 0.14 | 0.05 | 0.02 | 0.01 | 0.00 | 0.00 | 0.00 | 0.00 |
|  |  | **GLU** | **220** | 0.62 | 0.58 | 0.55 | 0.52 | 0.50 | 0.48 | 0.46 | 0.43 | 0.40 |
|  |  | **GLU** | **229** | 0.00 | 0.00 | 0.00 | 0.00 | 0.00 | 0.00 | 0.00 | 0.00 | 0.00 |
|  |  | **GLU** | **236** | 0.01 | 0.01 | 0.00 | 0.00 | 0.00 | 0.00 | 0.00 | 0.00 | 0.00 |
|  |  | **GLU** | **239** | 0.96 | 0.95 | 0.94 | 0.93 | 0.93 | 0.92 | 0.92 | 0.92 | 0.93 |
|  |  | **GLU** | **243** | 0.97 | 0.97 | 0.96 | 0.96 | 0.95 | 0.94 | 0.93 | 0.91 | 0.91 |
|  |  | **GLU** | **255** | 0.00 | 0.00 | 0.00 | 0.00 | 0.00 | 0.00 | 0.00 | 0.00 | 0.00 |
|  |  | **GLU** | **278** | 0.10 | 0.04 | 0.02 | 0.01 | 0.00 | 0.00 | 0.00 | 0.00 | 0.00 |
|  |  | **GLU** | **299** | 0.95 | 0.91 | 0.82 | 0.63 | 0.37 | 0.16 | 0.06 | 0.02 | 0.01 |
|  |  | **GLU** | **314** | 0.00 | 0.00 | 0.00 | 0.00 | 0.00 | 0.00 | 0.00 | 0.00 | 0.00 |
|  |  | **GLU** | **320** | 0.35 | 0.16 | 0.06 | 0.02 | 0.01 | 0.00 | 0.00 | 0.00 | 0.00 |
|  |  | **GLU** | **339** | 0.24 | 0.20 | 0.15 | 0.09 | 0.05 | 0.02 | 0.01 | 0.00 | 0.00 |
|  |  | **GLU** | **343** | 0.73 | 0.62 | 0.46 | 0.29 | 0.14 | 0.05 | 0.02 | 0.01 | 0.00 |
|  |  | **GLU** | **354** | 0.94 | 0.92 | 0.90 | 0.87 | 0.83 | 0.74 | 0.61 | 0.44 | 0.28 |
|  |  | **GLU** | **358** | 0.13 | 0.05 | 0.02 | 0.01 | 0.00 | 0.00 | 0.00 | 0.00 | 0.00 |
|  |  | **GLU** | **363** | 0.34 | 0.17 | 0.07 | 0.03 | 0.01 | 0.00 | 0.00 | 0.00 | 0.00 |
|  |  | **GLU** | **374** | 0.00 | 0.00 | 0.00 | 0.00 | 0.00 | 0.00 | 0.00 | 0.00 | 0.00 |
|  |  | **GLU** | **397** | 0.01 | 0.01 | 0.00 | 0.00 | 0.00 | 0.00 | 0.00 | 0.00 | 0.00 |
|  |  | **GLU** | **402** | 0.00 | 0.00 | 0.00 | 0.00 | 0.00 | 0.00 | 0.00 | 0.00 | 0.00 |
|  |  | **GLU** | **412** | 0.00 | 0.00 | 0.00 | 0.00 | 0.00 | 0.00 | 0.00 | 0.00 | 0.00 |
|  |  | **GLU** | **417** | 1.00 | 1.00 | 1.00 | 1.00 | 1.00 | 0.99 | 0.99 | 0.99 | 0.98 |
|  |  | **GLU** | **420** | 0.00 | 0.00 | 0.00 | 0.00 | 0.00 | 0.00 | 0.00 | 0.00 | 0.00 |
|  |  | **GLU** | **426** | 0.01 | 0.00 | 0.00 | 0.00 | 0.00 | 0.00 | 0.00 | 0.00 | 0.00 |
|  |  | **GLU** | **451** | 0.59 | 0.37 | 0.20 | 0.12 | 0.08 | 0.06 | 0.04 | 0.03 | 0.02 |
|  |  | **GLU** | **458** | 0.71 | 0.51 | 0.28 | 0.12 | 0.04 | 0.02 | 0.01 | 0.00 | 0.00 |
|  | **subunit B** | **GLU** | **80** | 1.00 | 1.00 | 1.00 | 1.00 | 1.00 | 1.00 | 1.00 | 1.00 | 0.99 |
|  |  | **GLU** | **98** | 0.98 | 0.98 | 0.98 | 0.97 | 0.97 | 0.97 | 0.97 | 0.98 | 0.98 |
|  |  | **GLU** | **114** | 0.26 | 0.15 | 0.08 | 0.04 | 0.02 | 0.01 | 0.00 | 0.00 | 0.00 |
|  |  | **GLU** | **124** | 0.14 | 0.05 | 0.02 | 0.01 | 0.00 | 0.00 | 0.00 | 0.00 | 0.00 |
|  |  | **GLU** | **133** | 0.33 | 0.18 | 0.10 | 0.05 | 0.02 | 0.01 | 0.00 | 0.00 | 0.00 |
|  |  | **GLU** | **137** | 0.87 | 0.76 | 0.56 | 0.31 | 0.13 | 0.05 | 0.02 | 0.01 | 0.00 |
|  |  | **GLU** | **157** | 0.00 | 0.00 | 0.00 | 0.00 | 0.00 | 0.00 | 0.00 | 0.00 | 0.00 |
|  |  | **GLU** | **168** | 0.00 | 0.00 | 0.00 | 0.00 | 0.00 | 0.00 | 0.00 | 0.00 | 0.00 |
|  |  | **GLU** | **178** | 0.20 | 0.08 | 0.03 | 0.01 | 0.01 | 0.00 | 0.00 | 0.00 | 0.00 |
|  |  | **GLU** | **183** | 0.01 | 0.00 | 0.00 | 0.00 | 0.00 | 0.00 | 0.00 | 0.00 | 0.00 |
|  |  | **GLU** | **220** | 1.00 | 1.00 | 0.99 | 0.99 | 0.99 | 0.98 | 0.98 | 0.97 | 0.97 |
|  |  | **GLU** | **229** | 0.00 | 0.00 | 0.00 | 0.00 | 0.00 | 0.00 | 0.00 | 0.00 | 0.00 |
|  |  | **GLU** | **236** | 0.00 | 0.00 | 0.00 | 0.00 | 0.00 | 0.00 | 0.00 | 0.00 | 0.00 |
|  |  | **GLU** | **239** | 0.97 | 0.96 | 0.95 | 0.94 | 0.92 | 0.92 | 0.91 | 0.91 | 0.91 |
|  |  | **GLU** | **243** | 0.95 | 0.95 | 0.94 | 0.93 | 0.92 | 0.91 | 0.89 | 0.87 | 0.86 |
|  |  | **GLU** | **255** | 0.00 | 0.00 | 0.00 | 0.00 | 0.00 | 0.00 | 0.00 | 0.00 | 0.00 |
|  |  | **GLU** | **278** | 0.74 | 0.56 | 0.37 | 0.22 | 0.13 | 0.08 | 0.04 | 0.02 | 0.01 |
|  |  | **GLU** | **299** | 0.59 | 0.40 | 0.26 | 0.18 | 0.15 | 0.12 | 0.09 | 0.08 | 0.05 |
|  |  | **GLU** | **314** | 0.00 | 0.00 | 0.00 | 0.00 | 0.00 | 0.00 | 0.00 | 0.00 | 0.00 |
|  |  | **GLU** | **320** | 0.27 | 0.12 | 0.04 | 0.02 | 0.01 | 0.00 | 0.00 | 0.00 | 0.00 |
|  |  | **GLU** | **339** | 0.17 | 0.13 | 0.10 | 0.06 | 0.03 | 0.01 | 0.01 | 0.00 | 0.00 |
|  |  | **GLU** | **343** | 0.36 | 0.20 | 0.10 | 0.04 | 0.02 | 0.01 | 0.00 | 0.00 | 0.00 |
|  |  | **GLU** | **354** | 0.86 | 0.82 | 0.76 | 0.68 | 0.60 | 0.49 | 0.37 | 0.28 | 0.21 |
|  |  | **GLU** | **358** | 0.17 | 0.07 | 0.02 | 0.01 | 0.00 | 0.00 | 0.00 | 0.00 | 0.00 |
|  |  | **GLU** | **363** | 0.10 | 0.04 | 0.02 | 0.01 | 0.00 | 0.00 | 0.00 | 0.00 | 0.00 |
|  |  | **GLU** | **374** | 0.00 | 0.00 | 0.00 | 0.00 | 0.00 | 0.00 | 0.00 | 0.00 | 0.00 |
|  |  | **GLU** | **397** | 0.00 | 0.00 | 0.00 | 0.00 | 0.00 | 0.00 | 0.00 | 0.00 | 0.00 |
|  |  | **GLU** | **402** | 0.04 | 0.02 | 0.01 | 0.00 | 0.00 | 0.00 | 0.00 | 0.00 | 0.00 |
|  |  | **GLU** | **412** | 0.00 | 0.00 | 0.00 | 0.00 | 0.00 | 0.00 | 0.00 | 0.00 | 0.00 |
|  |  | **GLU** | **417** | 1.00 | 1.00 | 1.00 | 0.99 | 0.99 | 0.99 | 0.99 | 0.98 | 0.97 |
|  |  | **GLU** | **420** | 0.04 | 0.02 | 0.01 | 0.00 | 0.00 | 0.00 | 0.00 | 0.00 | 0.00 |
|  |  | **GLU** | **426** | 0.00 | 0.00 | 0.00 | 0.00 | 0.00 | 0.00 | 0.00 | 0.00 | 0.00 |
|  |  | **GLU** | **451** | 0.01 | 0.00 | 0.00 | 0.00 | 0.00 | 0.00 | 0.00 | 0.00 | 0.00 |
|  |  | **GLU** | **458** | 0.17 | 0.08 | 0.04 | 0.02 | 0.01 | 0.00 | 0.00 | 0.00 | 0.00 |
|  | **subunit C** | **GLU** | **80** | 1.00 | 1.00 | 1.00 | 1.00 | 1.00 | 1.00 | 0.99 | 0.99 | 0.98 |
|  |  | **GLU** | **98** | 0.99 | 0.98 | 0.98 | 0.97 | 0.96 | 0.95 | 0.96 | 0.96 | 0.97 |
|  |  | **GLU** | **114** | 0.60 | 0.41 | 0.23 | 0.11 | 0.05 | 0.02 | 0.01 | 0.00 | 0.00 |
|  |  | **GLU** | **124** | 0.05 | 0.02 | 0.01 | 0.00 | 0.00 | 0.00 | 0.00 | 0.00 | 0.00 |
|  |  | **GLU** | **133** | 0.65 | 0.43 | 0.22 | 0.09 | 0.03 | 0.01 | 0.00 | 0.00 | 0.00 |
|  |  | **GLU** | **137** | 0.35 | 0.20 | 0.10 | 0.04 | 0.01 | 0.00 | 0.00 | 0.00 | 0.00 |
|  |  | **GLU** | **157** | 0.00 | 0.00 | 0.00 | 0.00 | 0.00 | 0.00 | 0.00 | 0.00 | 0.00 |
|  |  | **GLU** | **168** | 0.01 | 0.01 | 0.00 | 0.00 | 0.00 | 0.00 | 0.00 | 0.00 | 0.00 |
|  |  | **GLU** | **178** | 0.04 | 0.02 | 0.01 | 0.00 | 0.00 | 0.00 | 0.00 | 0.00 | 0.00 |
|  |  | **GLU** | **183** | 0.00 | 0.00 | 0.00 | 0.00 | 0.00 | 0.00 | 0.00 | 0.00 | 0.00 |
|  |  | **GLU** | **220** | 0.75 | 0.71 | 0.67 | 0.63 | 0.59 | 0.56 | 0.54 | 0.53 | 0.51 |
|  |  | **GLU** | **229** | 0.00 | 0.00 | 0.00 | 0.00 | 0.00 | 0.00 | 0.00 | 0.00 | 0.00 |
|  |  | **GLU** | **236** | 0.00 | 0.00 | 0.00 | 0.00 | 0.00 | 0.00 | 0.00 | 0.00 | 0.00 |
|  |  | **GLU** | **239** | 1.00 | 1.00 | 1.00 | 1.00 | 1.00 | 1.00 | 1.00 | 1.00 | 1.00 |
|  |  | **GLU** | **243** | 0.02 | 0.01 | 0.01 | 0.01 | 0.01 | 0.00 | 0.00 | 0.00 | 0.00 |
|  |  | **GLU** | **255** | 0.00 | 0.00 | 0.00 | 0.00 | 0.00 | 0.00 | 0.00 | 0.00 | 0.00 |
|  |  | **GLU** | **278** | 0.00 | 0.00 | 0.00 | 0.00 | 0.00 | 0.00 | 0.00 | 0.00 | 0.00 |
|  |  | **GLU** | **299** | 0.98 | 0.95 | 0.88 | 0.73 | 0.48 | 0.24 | 0.09 | 0.03 | 0.01 |
|  |  | **GLU** | **314** | 0.00 | 0.00 | 0.00 | 0.00 | 0.00 | 0.00 | 0.00 | 0.00 | 0.00 |
|  |  | **GLU** | **320** | 0.33 | 0.15 | 0.06 | 0.02 | 0.01 | 0.00 | 0.00 | 0.00 | 0.00 |
|  |  | **GLU** | **339** | 0.15 | 0.13 | 0.09 | 0.04 | 0.02 | 0.01 | 0.00 | 0.00 | 0.00 |
|  |  | **GLU** | **343** | 0.25 | 0.13 | 0.06 | 0.03 | 0.01 | 0.00 | 0.00 | 0.00 | 0.00 |
|  |  | **GLU** | **354** | 0.96 | 0.92 | 0.84 | 0.73 | 0.62 | 0.50 | 0.39 | 0.27 | 0.16 |
|  |  | **GLU** | **358** | 0.16 | 0.07 | 0.03 | 0.01 | 0.00 | 0.00 | 0.00 | 0.00 | 0.00 |
|  |  | **GLU** | **363** | 0.41 | 0.24 | 0.11 | 0.05 | 0.02 | 0.01 | 0.00 | 0.00 | 0.00 |
|  |  | **GLU** | **374** | 0.00 | 0.00 | 0.00 | 0.00 | 0.00 | 0.00 | 0.00 | 0.00 | 0.00 |
|  |  | **GLU** | **397** | 0.31 | 0.20 | 0.11 | 0.05 | 0.02 | 0.01 | 0.00 | 0.00 | 0.00 |
|  |  | **GLU** | **402** | 0.00 | 0.00 | 0.00 | 0.00 | 0.00 | 0.00 | 0.00 | 0.00 | 0.00 |
|  |  | **GLU** | **412** | 0.00 | 0.00 | 0.00 | 0.00 | 0.00 | 0.00 | 0.00 | 0.00 | 0.00 |
|  |  | **GLU** | **417** | 1.00 | 1.00 | 1.00 | 1.00 | 1.00 | 1.00 | 0.99 | 0.99 | 0.98 |
|  |  | **GLU** | **420** | 0.03 | 0.01 | 0.01 | 0.00 | 0.00 | 0.00 | 0.00 | 0.00 | 0.00 |
|  |  | **GLU** | **426** | 0.01 | 0.00 | 0.00 | 0.00 | 0.00 | 0.00 | 0.00 | 0.00 | 0.00 |
|  |  | **GLU** | **451** | 0.97 | 0.96 | 0.94 | 0.92 | 0.89 | 0.81 | 0.67 | 0.47 | 0.25 |
|  | **subunit A** | **LYS** | **43** | 1.00 | 1.00 | 1.00 | 1.00 | 1.00 | 1.00 | 0.99 | 0.98 | 0.94 |
|  |  | **LYS** | **77** | 1.00 | 0.99 | 0.98 | 0.96 | 0.93 | 0.87 | 0.77 | 0.63 | 0.47 |
|  |  | **LYS** | **106** | 1.00 | 1.00 | 1.00 | 1.00 | 1.00 | 1.00 | 1.00 | 0.99 | 0.97 |
|  |  | **LYS** | **134** | 1.00 | 1.00 | 1.00 | 1.00 | 1.00 | 1.00 | 1.00 | 1.00 | 0.99 |
|  |  | **LYS** | **142** | 1.00 | 1.00 | 1.00 | 1.00 | 1.00 | 1.00 | 0.99 | 0.97 | 0.92 |
|  |  | **LYS** | **149** | 1.00 | 1.00 | 1.00 | 1.00 | 1.00 | 1.00 | 0.99 | 0.98 | 0.94 |
|  |  | **LYS** | **151** | 1.00 | 1.00 | 1.00 | 1.00 | 1.00 | 0.99 | 0.98 | 0.95 | 0.86 |
|  |  | **LYS** | **186** | 1.00 | 1.00 | 1.00 | 1.00 | 1.00 | 1.00 | 1.00 | 0.99 | 0.96 |
|  |  | **LYS** | **194** | 1.00 | 1.00 | 1.00 | 1.00 | 1.00 | 1.00 | 1.00 | 1.00 | 1.00 |
|  |  | **LYS** | **205** | 1.00 | 1.00 | 1.00 | 1.00 | 1.00 | 1.00 | 1.00 | 1.00 | 0.99 |
|  |  | **LYS** | **212** | 1.00 | 1.00 | 1.00 | 1.00 | 1.00 | 1.00 | 1.00 | 1.00 | 1.00 |
|  |  | **LYS** | **247** | 1.00 | 1.00 | 1.00 | 1.00 | 1.00 | 1.00 | 1.00 | 1.00 | 0.99 |
|  |  | **LYS** | **292** | 1.00 | 1.00 | 1.00 | 1.00 | 1.00 | 1.00 | 1.00 | 1.00 | 1.00 |
|  |  | **LYS** | **342** | 1.00 | 1.00 | 1.00 | 1.00 | 1.00 | 1.00 | 1.00 | 1.00 | 1.00 |
|  |  | **LYS** | **355** | 1.00 | 1.00 | 1.00 | 1.00 | 1.00 | 1.00 | 1.00 | 1.00 | 1.00 |
|  |  | **LYS** | **373** | 1.00 | 1.00 | 1.00 | 1.00 | 1.00 | 1.00 | 1.00 | 1.00 | 1.00 |
|  |  | **LYS** | **379** | 1.00 | 1.00 | 1.00 | 1.00 | 0.99 | 0.99 | 0.97 | 0.93 | 0.86 |
|  |  | **LYS** | **383** | 1.00 | 1.00 | 1.00 | 1.00 | 1.00 | 1.00 | 1.00 | 1.00 | 1.00 |
|  |  | **LYS** | **387** | 1.00 | 1.00 | 1.00 | 1.00 | 1.00 | 1.00 | 1.00 | 1.00 | 1.00 |
|  |  | **LYS** | **391** | 1.00 | 1.00 | 1.00 | 1.00 | 1.00 | 1.00 | 1.00 | 1.00 | 0.98 |
|  |  | **LYS** | **392** | 1.00 | 1.00 | 1.00 | 1.00 | 1.00 | 1.00 | 1.00 | 1.00 | 1.00 |
|  |  | **LYS** | **395** | 1.00 | 1.00 | 1.00 | 1.00 | 1.00 | 1.00 | 1.00 | 1.00 | 1.00 |
|  |  | **LYS** | **422** | 1.00 | 1.00 | 1.00 | 1.00 | 1.00 | 1.00 | 1.00 | 1.00 | 1.00 |
|  |  | **LYS** | **423** | 1.00 | 1.00 | 1.00 | 1.00 | 1.00 | 1.00 | 0.99 | 0.97 | 0.92 |
|  | **subunit B** | **LYS** | **43** | 1.00 | 0.99 | 0.98 | 0.95 | 0.88 | 0.72 | 0.48 | 0.24 | 0.10 |
|  |  | **LYS** | **77** | 1.00 | 0.99 | 0.99 | 0.97 | 0.95 | 0.92 | 0.87 | 0.77 | 0.64 |
|  |  | **LYS** | **106** | 1.00 | 1.00 | 1.00 | 1.00 | 1.00 | 1.00 | 1.00 | 1.00 | 0.99 |
|  |  | **LYS** | **134** | 1.00 | 1.00 | 1.00 | 1.00 | 1.00 | 1.00 | 1.00 | 1.00 | 0.98 |
|  |  | **LYS** | **142** | 1.00 | 1.00 | 1.00 | 1.00 | 1.00 | 1.00 | 0.99 | 0.98 | 0.96 |
|  |  | **LYS** | **149** | 1.00 | 1.00 | 1.00 | 1.00 | 1.00 | 1.00 | 1.00 | 0.99 | 0.98 |
|  |  | **LYS** | **151** | 1.00 | 1.00 | 1.00 | 0.99 | 0.98 | 0.96 | 0.88 | 0.73 | 0.47 |
|  |  | **LYS** | **186** | 1.00 | 1.00 | 1.00 | 1.00 | 1.00 | 1.00 | 1.00 | 1.00 | 1.00 |
|  |  | **LYS** | **194** | 1.00 | 1.00 | 1.00 | 1.00 | 1.00 | 1.00 | 1.00 | 1.00 | 1.00 |
|  |  | **LYS** | **205** | 1.00 | 1.00 | 1.00 | 1.00 | 1.00 | 1.00 | 1.00 | 1.00 | 1.00 |
|  |  | **LYS** | **212** | 1.00 | 1.00 | 1.00 | 1.00 | 1.00 | 1.00 | 1.00 | 1.00 | 1.00 |
|  |  | **LYS** | **247** | 1.00 | 1.00 | 1.00 | 1.00 | 1.00 | 1.00 | 1.00 | 1.00 | 1.00 |
|  |  | **LYS** | **292** | 1.00 | 1.00 | 1.00 | 1.00 | 1.00 | 1.00 | 1.00 | 1.00 | 1.00 |
|  |  | **LYS** | **342** | 1.00 | 1.00 | 1.00 | 1.00 | 1.00 | 1.00 | 1.00 | 1.00 | 1.00 |
|  |  | **LYS** | **355** | 1.00 | 1.00 | 1.00 | 1.00 | 1.00 | 1.00 | 1.00 | 1.00 | 1.00 |
|  |  | **LYS** | **373** | 1.00 | 1.00 | 1.00 | 1.00 | 1.00 | 1.00 | 1.00 | 1.00 | 1.00 |
|  |  | **LYS** | **379** | 1.00 | 1.00 | 1.00 | 1.00 | 1.00 | 1.00 | 0.99 | 0.99 | 0.98 |
|  |  | **LYS** | **383** | 1.00 | 1.00 | 1.00 | 1.00 | 1.00 | 1.00 | 1.00 | 1.00 | 1.00 |
|  |  | **LYS** | **387** | 1.00 | 1.00 | 1.00 | 1.00 | 1.00 | 1.00 | 1.00 | 1.00 | 1.00 |
|  |  | **LYS** | **391** | 1.00 | 1.00 | 1.00 | 1.00 | 1.00 | 1.00 | 1.00 | 1.00 | 0.99 |
|  |  | **LYS** | **392** | 1.00 | 1.00 | 1.00 | 1.00 | 1.00 | 1.00 | 1.00 | 1.00 | 1.00 |
|  |  | **LYS** | **395** | 1.00 | 1.00 | 1.00 | 1.00 | 1.00 | 1.00 | 1.00 | 1.00 | 1.00 |
|  |  | **LYS** | **422** | 1.00 | 1.00 | 1.00 | 1.00 | 1.00 | 1.00 | 1.00 | 1.00 | 0.99 |
|  |  | **LYS** | **423** | 1.00 | 1.00 | 1.00 | 1.00 | 1.00 | 1.00 | 0.99 | 0.99 | 0.96 |
|  |  | **LYS** | **461** | 1.00 | 1.00 | 1.00 | 1.00 | 1.00 | 1.00 | 1.00 | 1.00 | 0.99 |
|  | **subunit C** | **LYS** | **43** | 1.00 | 1.00 | 1.00 | 1.00 | 1.00 | 1.00 | 1.00 | 1.00 | 1.00 |
|  |  | **LYS** | **77** | 1.00 | 1.00 | 0.99 | 0.99 | 0.98 | 0.96 | 0.91 | 0.83 | 0.72 |
|  |  | **LYS** | **106** | 1.00 | 1.00 | 1.00 | 1.00 | 1.00 | 1.00 | 1.00 | 1.00 | 0.99 |
|  |  | **LYS** | **134** | 1.00 | 1.00 | 1.00 | 1.00 | 1.00 | 0.99 | 0.99 | 0.97 | 0.92 |
|  |  | **LYS** | **142** | 1.00 | 1.00 | 1.00 | 1.00 | 1.00 | 1.00 | 0.99 | 0.99 | 0.97 |
|  |  | **LYS** | **149** | 1.00 | 1.00 | 1.00 | 1.00 | 1.00 | 0.99 | 0.97 | 0.92 | 0.80 |
|  |  | **LYS** | **151** | 1.00 | 1.00 | 1.00 | 1.00 | 1.00 | 1.00 | 0.99 | 0.97 | 0.93 |
|  |  | **LYS** | **186** | 1.00 | 1.00 | 1.00 | 1.00 | 1.00 | 0.99 | 0.99 | 0.97 | 0.92 |
|  |  | **LYS** | **194** | 1.00 | 1.00 | 1.00 | 1.00 | 1.00 | 1.00 | 1.00 | 1.00 | 1.00 |
|  |  | **LYS** | **205** | 1.00 | 1.00 | 1.00 | 1.00 | 1.00 | 1.00 | 1.00 | 0.99 | 0.97 |
|  |  | **LYS** | **212** | 1.00 | 1.00 | 1.00 | 1.00 | 1.00 | 1.00 | 1.00 | 1.00 | 1.00 |
|  |  | **LYS** | **247** | 1.00 | 1.00 | 1.00 | 1.00 | 1.00 | 1.00 | 1.00 | 1.00 | 1.00 |
|  |  | **LYS** | **292** | 1.00 | 1.00 | 1.00 | 1.00 | 1.00 | 1.00 | 1.00 | 1.00 | 1.00 |
|  |  | **LYS** | **342** | 1.00 | 1.00 | 1.00 | 1.00 | 1.00 | 1.00 | 1.00 | 1.00 | 1.00 |
|  |  | **LYS** | **355** | 1.00 | 1.00 | 1.00 | 1.00 | 1.00 | 1.00 | 1.00 | 1.00 | 1.00 |
|  |  | **LYS** | **373** | 1.00 | 1.00 | 1.00 | 1.00 | 1.00 | 1.00 | 1.00 | 1.00 | 1.00 |
|  |  | **LYS** | **379** | 1.00 | 1.00 | 1.00 | 1.00 | 1.00 | 1.00 | 1.00 | 1.00 | 0.99 |
|  |  | **LYS** | **383** | 1.00 | 1.00 | 1.00 | 1.00 | 1.00 | 1.00 | 0.99 | 0.99 | 0.97 |
|  |  | **LYS** | **387** | 1.00 | 1.00 | 0.99 | 0.99 | 0.97 | 0.92 | 0.80 | 0.59 | 0.35 |
|  |  | **LYS** | **391** | 1.00 | 1.00 | 1.00 | 1.00 | 1.00 | 0.99 | 0.97 | 0.93 | 0.87 |
|  |  | **LYS** | **392** | 1.00 | 1.00 | 1.00 | 1.00 | 1.00 | 1.00 | 1.00 | 1.00 | 1.00 |
|  |  | **LYS** | **395** | 1.00 | 1.00 | 1.00 | 1.00 | 1.00 | 1.00 | 1.00 | 1.00 | 1.00 |
|  |  | **LYS** | **422** | 1.00 | 1.00 | 1.00 | 1.00 | 1.00 | 1.00 | 1.00 | 0.99 | 0.99 |
|  |  | **LYS** | **423** | 1.00 | 1.00 | 1.00 | 1.00 | 1.00 | 1.00 | 1.00 | 0.99 | 0.96 |
|  | **subunit A** | **TYR** | **68** | 1.00 | 1.00 | 1.00 | 1.00 | 1.00 | 1.00 | 1.00 | 1.00 | 0.99 |
|  |  | **TYR** | **69** | 1.00 | 1.00 | 1.00 | 1.00 | 1.00 | 1.00 | 1.00 | 1.00 | 1.00 |
|  |  | **TYR** | **72** | 1.00 | 1.00 | 1.00 | 1.00 | 1.00 | 1.00 | 1.00 | 1.00 | 1.00 |
|  |  | **TYR** | **110** | 1.00 | 1.00 | 1.00 | 1.00 | 1.00 | 1.00 | 1.00 | 1.00 | 1.00 |
|  |  | **TYR** | **123** | 1.00 | 1.00 | 1.00 | 1.00 | 1.00 | 1.00 | 1.00 | 1.00 | 1.00 |
|  |  | **TYR** | **159** | 1.00 | 1.00 | 1.00 | 1.00 | 1.00 | 1.00 | 1.00 | 1.00 | 1.00 |
|  |  | **TYR** | **192** | 1.00 | 1.00 | 1.00 | 1.00 | 1.00 | 1.00 | 1.00 | 1.00 | 1.00 |
|  |  | **TYR** | **196** | 1.00 | 1.00 | 1.00 | 1.00 | 1.00 | 1.00 | 1.00 | 1.00 | 1.00 |
|  |  | **TYR** | **230** | 1.00 | 1.00 | 1.00 | 1.00 | 1.00 | 1.00 | 1.00 | 1.00 | 1.00 |
|  |  | **TYR** | **283** | 1.00 | 1.00 | 1.00 | 1.00 | 1.00 | 1.00 | 1.00 | 1.00 | 1.00 |
|  |  | **TYR** | **301** | 1.00 | 1.00 | 1.00 | 1.00 | 1.00 | 1.00 | 1.00 | 1.00 | 1.00 |
|  |  | **TYR** | **304** | 1.00 | 1.00 | 1.00 | 1.00 | 1.00 | 1.00 | 1.00 | 1.00 | 1.00 |
|  |  | **TYR** | **317** | 1.00 | 1.00 | 1.00 | 1.00 | 1.00 | 1.00 | 1.00 | 1.00 | 1.00 |
|  |  | **TYR** | **335** | 1.00 | 1.00 | 1.00 | 1.00 | 1.00 | 1.00 | 1.00 | 1.00 | 1.00 |
|  |  | **TYR** | **341** | 1.00 | 1.00 | 1.00 | 1.00 | 1.00 | 1.00 | 1.00 | 1.00 | 1.00 |
|  |  | **TYR** | **359** | 1.00 | 1.00 | 1.00 | 1.00 | 1.00 | 1.00 | 1.00 | 0.99 | 0.97 |
|  |  | **TYR** | **371** | 1.00 | 1.00 | 1.00 | 1.00 | 1.00 | 1.00 | 1.00 | 1.00 | 1.00 |
|  |  | **TYR** | **388** | 1.00 | 1.00 | 1.00 | 1.00 | 1.00 | 1.00 | 1.00 | 1.00 | 1.00 |
|  |  | **TYR** | **393** | 1.00 | 1.00 | 1.00 | 1.00 | 1.00 | 1.00 | 1.00 | 1.00 | 1.00 |
|  |  | **TYR** | **399** | 1.00 | 1.00 | 1.00 | 1.00 | 1.00 | 1.00 | 1.00 | 1.00 | 1.00 |
|  |  | **TYR** | **416** | 1.00 | 1.00 | 1.00 | 1.00 | 1.00 | 1.00 | 1.00 | 1.00 | 1.00 |
|  |  | **TYR** | **425** | 1.00 | 1.00 | 1.00 | 1.00 | 1.00 | 1.00 | 1.00 | 1.00 | 1.00 |
|  |  | **TYR** | **455** | 1.00 | 1.00 | 1.00 | 1.00 | 1.00 | 1.00 | 0.99 | 0.98 | 0.93 |
|  |  | **TYR** | **457** | 1.00 | 1.00 | 1.00 | 1.00 | 1.00 | 1.00 | 1.00 | 0.99 | 0.99 |
|  | **subunit B** | **TYR** | **68** | 1.00 | 1.00 | 1.00 | 1.00 | 1.00 | 1.00 | 1.00 | 0.99 | 0.99 |
|  |  | **TYR** | **69** | 1.00 | 1.00 | 1.00 | 1.00 | 1.00 | 1.00 | 1.00 | 1.00 | 1.00 |
|  |  | **TYR** | **72** | 1.00 | 1.00 | 1.00 | 1.00 | 1.00 | 1.00 | 1.00 | 1.00 | 1.00 |
|  |  | **TYR** | **110** | 1.00 | 1.00 | 1.00 | 1.00 | 1.00 | 1.00 | 1.00 | 1.00 | 1.00 |
|  |  | **TYR** | **123** | 1.00 | 1.00 | 1.00 | 1.00 | 1.00 | 1.00 | 1.00 | 1.00 | 1.00 |
|  |  | **TYR** | **159** | 1.00 | 1.00 | 1.00 | 1.00 | 1.00 | 1.00 | 1.00 | 1.00 | 1.00 |
|  |  | **TYR** | **192** | 1.00 | 1.00 | 1.00 | 1.00 | 1.00 | 1.00 | 1.00 | 1.00 | 1.00 |
|  |  | **TYR** | **196** | 1.00 | 1.00 | 1.00 | 1.00 | 1.00 | 1.00 | 1.00 | 1.00 | 1.00 |
|  |  | **TYR** | **230** | 1.00 | 1.00 | 1.00 | 1.00 | 1.00 | 1.00 | 1.00 | 1.00 | 1.00 |
|  |  | **TYR** | **283** | 1.00 | 1.00 | 1.00 | 1.00 | 1.00 | 1.00 | 1.00 | 1.00 | 1.00 |
|  |  | **TYR** | **301** | 1.00 | 1.00 | 1.00 | 1.00 | 1.00 | 1.00 | 1.00 | 1.00 | 1.00 |
|  |  | **TYR** | **304** | 1.00 | 1.00 | 1.00 | 1.00 | 1.00 | 1.00 | 1.00 | 1.00 | 1.00 |
|  |  | **TYR** | **317** | 1.00 | 1.00 | 1.00 | 1.00 | 1.00 | 1.00 | 1.00 | 1.00 | 1.00 |
|  |  | **TYR** | **335** | 1.00 | 1.00 | 1.00 | 1.00 | 1.00 | 1.00 | 1.00 | 1.00 | 1.00 |
|  |  | **TYR** | **341** | 1.00 | 1.00 | 1.00 | 1.00 | 1.00 | 1.00 | 1.00 | 1.00 | 1.00 |
|  |  | **TYR** | **359** | 1.00 | 1.00 | 1.00 | 1.00 | 1.00 | 1.00 | 1.00 | 1.00 | 0.99 |
|  |  | **TYR** | **371** | 1.00 | 1.00 | 1.00 | 1.00 | 1.00 | 1.00 | 1.00 | 1.00 | 1.00 |
|  |  | **TYR** | **388** | 1.00 | 1.00 | 1.00 | 1.00 | 1.00 | 1.00 | 1.00 | 1.00 | 1.00 |
|  |  | **TYR** | **393** | 1.00 | 1.00 | 1.00 | 1.00 | 1.00 | 1.00 | 1.00 | 1.00 | 1.00 |
|  |  | **TYR** | **399** | 1.00 | 1.00 | 1.00 | 1.00 | 1.00 | 1.00 | 1.00 | 1.00 | 1.00 |
|  |  | **TYR** | **416** | 1.00 | 1.00 | 1.00 | 1.00 | 1.00 | 1.00 | 1.00 | 1.00 | 1.00 |
|  |  | **TYR** | **425** | 1.00 | 1.00 | 1.00 | 1.00 | 1.00 | 1.00 | 1.00 | 1.00 | 1.00 |
|  |  | **TYR** | **455** | 1.00 | 1.00 | 1.00 | 1.00 | 1.00 | 1.00 | 1.00 | 1.00 | 0.99 |
|  |  | **TYR** | **457** | 1.00 | 1.00 | 1.00 | 1.00 | 1.00 | 1.00 | 1.00 | 0.99 | 0.98 |
|  | **subunit C** | **TYR** | **68** | 1.00 | 1.00 | 1.00 | 1.00 | 1.00 | 1.00 | 1.00 | 1.00 | 0.99 |
|  |  | **TYR** | **69** | 1.00 | 1.00 | 1.00 | 1.00 | 1.00 | 1.00 | 1.00 | 1.00 | 1.00 |
|  |  | **TYR** | **72** | 1.00 | 1.00 | 1.00 | 1.00 | 1.00 | 1.00 | 1.00 | 1.00 | 1.00 |
|  |  | **TYR** | **110** | 1.00 | 1.00 | 1.00 | 1.00 | 1.00 | 1.00 | 1.00 | 1.00 | 0.99 |
|  |  | **TYR** | **123** | 1.00 | 1.00 | 1.00 | 1.00 | 1.00 | 0.99 | 0.97 | 0.92 | 0.79 |
|  |  | **TYR** | **159** | 1.00 | 1.00 | 1.00 | 1.00 | 1.00 | 1.00 | 1.00 | 1.00 | 1.00 |
|  |  | **TYR** | **192** | 1.00 | 1.00 | 1.00 | 1.00 | 1.00 | 1.00 | 1.00 | 1.00 | 1.00 |
|  |  | **TYR** | **196** | 1.00 | 1.00 | 1.00 | 1.00 | 1.00 | 1.00 | 1.00 | 1.00 | 1.00 |
|  |  | **TYR** | **230** | 1.00 | 1.00 | 1.00 | 1.00 | 1.00 | 1.00 | 1.00 | 1.00 | 1.00 |
|  |  | **TYR** | **283** | 1.00 | 1.00 | 1.00 | 1.00 | 1.00 | 1.00 | 1.00 | 1.00 | 1.00 |
|  |  | **TYR** | **301** | 1.00 | 1.00 | 1.00 | 1.00 | 1.00 | 1.00 | 1.00 | 1.00 | 1.00 |
|  |  | **TYR** | **304** | 1.00 | 1.00 | 1.00 | 1.00 | 1.00 | 1.00 | 1.00 | 1.00 | 1.00 |
|  |  | **TYR** | **317** | 1.00 | 1.00 | 1.00 | 1.00 | 1.00 | 1.00 | 1.00 | 1.00 | 1.00 |
|  |  | **TYR** | **335** | 1.00 | 1.00 | 1.00 | 1.00 | 1.00 | 1.00 | 1.00 | 1.00 | 1.00 |
|  |  | **TYR** | **341** | 1.00 | 1.00 | 1.00 | 1.00 | 1.00 | 1.00 | 1.00 | 1.00 | 1.00 |
|  |  | **TYR** | **359** | 1.00 | 1.00 | 1.00 | 1.00 | 1.00 | 1.00 | 1.00 | 0.99 | 0.97 |
|  |  | **TYR** | **371** | 1.00 | 1.00 | 1.00 | 1.00 | 1.00 | 1.00 | 1.00 | 1.00 | 1.00 |
|  |  | **TYR** | **388** | 1.00 | 1.00 | 1.00 | 1.00 | 1.00 | 1.00 | 1.00 | 1.00 | 1.00 |
|  |  | **TYR** | **393** | 1.00 | 1.00 | 1.00 | 1.00 | 1.00 | 1.00 | 1.00 | 1.00 | 1.00 |
|  |  | **TYR** | **399** | 1.00 | 1.00 | 1.00 | 1.00 | 1.00 | 1.00 | 1.00 | 1.00 | 1.00 |
|  |  | **TYR** | **416** | 1.00 | 1.00 | 1.00 | 1.00 | 1.00 | 1.00 | 1.00 | 1.00 | 1.00 |
|  |  | **TYR** | **425** | 1.00 | 1.00 | 1.00 | 1.00 | 1.00 | 1.00 | 1.00 | 1.00 | 1.00 |
|  |  | **TYR** | **455** | 1.00 | 1.00 | 1.00 | 1.00 | 1.00 | 0.99 | 0.98 | 0.94 | 0.86 |
|  |  | **TYR** | **457** | 1.00 | 1.00 | 1.00 | 1.00 | 1.00 | 0.99 | 0.98 | 0.95 | 0.89 |
|  | **subunit A** | **N-ter** | **42** | 0.99 | 0.98 | 0.95 | 0.87 | 0.69 | 0.44 | 0.24 | 0.11 | 0.05 |
|  |  | **C-ter** | **458** | 0.29 | 0.16 | 0.08 | 0.03 | 0.01 | 0.01 | 0.00 | 0.00 | 0.00 |
|  | **subunit B** | **N-ter** | **42** | 0.97 | 0.93 | 0.82 | 0.61 | 0.36 | 0.16 | 0.06 | 0.02 | 0.01 |
|  |  | **C-ter** | **461** | 0.89 | 0.76 | 0.53 | 0.28 | 0.12 | 0.04 | 0.02 | 0.00 | 0.00 |
|  | **subunit C** | **N-ter** | **40** | 0.79 | 0.60 | 0.38 | 0.23 | 0.13 | 0.06 | 0.03 | 0.01 | 0.00 |
|  |  | **C-ter** | **457** | 1.00 | 1.00 | 0.99 | 0.97 | 0.91 | 0.81 | 0.61 | 0.38 | 0.20 |
|  | **subunit A** | **HIS** | **74** | 0.82 | 0.66 | 0.42 | 0.22 | 0.10 | 0.04 | 0.02 | 0.01 | 0.01 |
|  |  | **HIS** | **111** | 0.79 | 0.62 | 0.40 | 0.21 | 0.09 | 0.03 | 0.01 | 0.00 | 0.00 |
|  |  | **HIS** | **164** | 0.98 | 0.98 | 0.98 | 0.97 | 0.95 | 0.91 | 0.82 | 0.67 | 0.50 |
|  |  | **HIS** | **251** | 1.00 | 1.00 | 1.00 | 1.00 | 1.00 | 1.00 | 0.99 | 0.98 | 0.96 |
|  |  | **HIS** | **328** | 0.00 | 0.00 | 0.00 | 0.00 | 0.00 | 0.00 | 0.00 | 0.00 | 0.00 |
|  | **subunit B** | **HIS** | **74** | 0.98 | 0.96 | 0.91 | 0.80 | 0.61 | 0.39 | 0.21 | 0.11 | 0.06 |
|  |  | **HIS** | **111** | 0.88 | 0.79 | 0.63 | 0.41 | 0.21 | 0.09 | 0.04 | 0.02 | 0.01 |
|  |  | **HIS** | **164** | 0.96 | 0.95 | 0.95 | 0.94 | 0.91 | 0.85 | 0.76 | 0.60 | 0.42 |
|  |  | **HIS** | **251** | 0.93 | 0.89 | 0.82 | 0.71 | 0.55 | 0.36 | 0.21 | 0.12 | 0.07 |
|  |  | **HIS** | **328** | 0.00 | 0.00 | 0.00 | 0.00 | 0.00 | 0.00 | 0.00 | 0.00 | 0.00 |
|  | **subunit C** | **HIS** | **74** | 0.95 | 0.88 | 0.76 | 0.56 | 0.34 | 0.17 | 0.09 | 0.04 | 0.02 |
|  |  | **HIS** | **111** | 0.64 | 0.46 | 0.27 | 0.13 | 0.05 | 0.02 | 0.01 | 0.00 | 0.00 |
|  |  | **HIS** | **164** | 0.90 | 0.88 | 0.87 | 0.85 | 0.80 | 0.70 | 0.55 | 0.39 | 0.24 |
|  |  | **HIS** | **251** | 0.98 | 0.95 | 0.90 | 0.81 | 0.66 | 0.48 | 0.30 | 0.17 | 0.09 |
|  |  | **HIS** | **328** | 0.00 | 0.00 | 0.00 | 0.00 | 0.00 | 0.00 | 0.00 | 0.00 | 0.00 |
